# Mechanisms of Neuronal Damage in Acute Hepatic Porphyrias

**DOI:** 10.3390/diagnostics11122205

**Published:** 2021-11-26

**Authors:** Andrea Ricci, Elena Di Pierro, Matteo Marcacci, Paolo Ventura

**Affiliations:** 1 Internal Medicine Unit, Department of Medical and Surgical Science for Children and Adults, University of Modena e Reggio Emilia, 41124 Modena, Italy; andrewrk92@gmail.com (A.R.); marcacci.matteo@aou.mo.it (M.M.); 2 Dipartimento di Medicina Interna, Fondazione IRCSS Cà Granda Ospedale Maggiore Policlinico, 20122 Milan, Italy; elena.dipierro@unimi.it

**Keywords:** porphyria, acute hepatic porphyrias, aminolevulinic acid, porphobilinogen, heme, neuronal damage, polyneuropathy, nitric oxide synthase, tryptophan metabolism, pyridoxal phosphate

## Abstract

Porphyrias are a group of congenital and acquired diseases caused by an enzymatic impairment in the biosynthesis of heme. Depending on the specific enzyme involved, different types of porphyrias (i.e., chronic vs. acute, cutaneous vs. neurovisceral, hepatic vs. erythropoietic) are described, with different clinical presentations. Acute hepatic porphyrias (AHPs) are characterized by life-threatening acute neuro-visceral crises (acute porphyric attacks, APAs), featuring a wide range of neuropathic (central, peripheral, autonomic) manifestations. APAs are usually unleashed by external “porphyrinogenic” triggers, which are thought to cause an increased metabolic demand for heme. During APAs, the heme precursors δ-aminolevulinic acid (ALA) and porphobilinogen (PBG) accumulate in the bloodstream and urine. Even though several hypotheses have been developed to explain the protean clinical picture of APAs, the exact mechanism of neuronal damage in AHPs is still a matter of debate. In recent decades, a role has been proposed for oxidative damage caused by ALA, mitochondrial and synaptic ALA toxicity, dysfunction induced by relative heme deficiency on cytochromes and other hemeproteins (i.e., nitric oxide synthases), pyridoxal phosphate functional deficiency, derangements in the metabolic pathways of tryptophan, and other factors. Since the pathway leading to the biosynthesis of heme is inscribed into a complex network of interactions, which also includes some fundamental processes of basal metabolism, a disruption in any of the steps of this pathway is likely to have multiple pathogenic effects. Here, we aim to provide a comprehensive review of the current evidence regarding the mechanisms of neuronal damage in AHPs.

## 1. Introduction

Porphyrias are a group of congenital and acquired diseases characterized by an impairment of the heme biosynthetic pathway [1]. Depending on the distinct enzyme deficiency and the tissue-specific isoform involved, different kinds of porphyria are described, the main distinction being made between cutaneous (or non-acute) porphyrias (congenital erythropoietic porphyria, porphyria cutanea tarda, hepato-erythropoietic porphyria, and X-linked/erythropoietic protoporphyria), mainly featuring a clinical picture of cutaneous phototoxicity, and acute hepatic porphyrias (AHPs). The latter comprise ALA dehydratase deficiency porphyria (AlaD-P), acute intermittent porphyria (AIP), hereditary coproporphyria (HCP), and variegate porphyria (VP). Both AlaD-P and AIP present only with acute neurovisceral manifestations, whereas HCP and PV display neurovisceral as well as subacute photosensitivity symptoms [2].

From a clinical perspective, neurovisceral acute porphyric attacks (APAs) are the most dramatic manifestations of AHPs [2,3,4,5,6]; they are usually unleashed by external triggers, such as fasting, alcoholic intake, the luteal phase of menstrual cycles, or porphyrinogenic drugs [7] (antiepileptics, barbiturates, griseofulvin, hormonal replacement therapy, etc.) and display a wide range of neuropathic manifestations [2,3,4,5,8,9].

The involvement of the autonomic nervous system in the splanchnic district occurs in the form of abdominal pain, constipation, diarrhea, nausea, and/or vomiting. Tachycardia (or other arrhythmias), hypertension, postural hypotension, diaphoresis, and sphincter disturbances are also commonly reported. Peripheral neuropathy is usually present as an acute axonal motor neuropathy, characteristically starting at the upper limbs and proximal muscles (as compared to Guillain-Barré syndrome), often accompanied by sensory involvement. Somewhat similar to Guillain-Barré syndrome, respiratory paralysis (due to phrenic nerve involvement) or bulbar paralysis can be dreaded and potentially life-threatening complications. Central nervous system (CNS) disturbances may develop throughout the entire course of APAs, the most dramatic being encephalopathy of varying severity, seizures (complex partial, tonic-clonic, myoclonic, and absence), and hyponatremia due to syndrome of inappropriate secretion of antidiuretic hormone (SIADH); psychiatric symptoms may complete the picture, ranging from depression, irritability, hallucination, to overt psychosis or catatonia. In patients with symptomatic AHP, a wide spectrum of histopathologic (and, more recently, radiological) changes have been observed in almost every part of the central and peripheral nervous systems [4,10,11]. Between crises, AIP patients may present with hyperintense (unspecific) small, rounded white matter lesions in T_2_-weighted magnetic resonance images [12].

It is important to note that phenotype penetration in congenital AHPs is probably very low; a French study of the general population found a mutation rate of 1/1675 for the gene responsible for AIP (*HMBS* coding for hydroxymethylbilane synthase, the third enzyme of the heme biosynthetic pathway) [13], while the yearly incidence of acute porphyric attacks in Europe is estimated to be 0.5–1 over 100.000 [14]. Similarly, based on genomic/exomic database interrogations, a clinical penetrance of 1% was estimated for *HMBS* heterozygous mutations [15]. Early studies have attempted to link some mutations in genes that are causative of AHP with the presence of psychiatric illnesses in the absence of any other overt clinical manifestations [16,17,18]. Although interesting, these findings are debatable and should be reconfirmed by contemporary investigations that comply with today’s standards of diagnostic methodology.

During porphyric crises, increased levels of the heme precursors *δ*-amino levulinic acid (ALA) and porphobilinogen (PBG) are measured in urine, indicating an enhanced activity of liver ALA synthase (ALAS1), the first enzyme in the heme biosynthetic pathway [3,6]. ALAS1 is probably overexpressed as a result of greater demands for heme under “porphyrinogenic” stress conditions.

Some diseases, either congenital (i.e., hereditary tyrosinemia) or acquired (i.e., lead intoxication) present classically as AHP-like syndromes [19,20]. Type I hereditary tyrosinemia is a rare and inheritable metabolic disturbance of the phenylalanine metabolism, where, due to an enzymatic dysfunction (fumaryl-acetoacetate hydrolase deficiency), a build-up of tyrosine and succinylacetone (SA) occurs [19]. SA is a by-product of tyrosine breakdown, which inhibits *δ*-aminolevulinic acid dehydratase (ALAD), the second enzyme of the heme biosynthetic pathway [21], thus leading to the accumulation of ALA. Lead displaces a zinc ion from a zinc-cysteine coordination complex of ALAD, suppressing its activity by either steric hindrance or by catalytic impairment [22].

While the pathogenesis of cutaneous manifestations is chiefly attributed to the light-dependent release of cytotoxic reactive oxygen species (ROS) exerted by the type I/II photosensitized reactions induced by porphyrins (i.e., cyclic tetrapyrrolic heme precursors) [23,24], the exact mechanism underlying the neurologic impairment in AHP is still a matter of debate [4,5,25]. Despite some early evidence of direct porphobilinogen neurotoxicity [26], in recent decades, the majority of authors have focused on the occurrences of direct ALA neurotoxicity, neurologic damage following heme depletion, and a few others. The present work aims to provide a comprehensive review of the current evidence regarding the principal hypotheses for neuronal damage in AHPs.

### Experimental Models for Studying AHPs

Several experimental models have helped to elucidate the pathophysiology of neurologic damage in AHPs. 

As previously mentioned, succinyl acetone (SA) inhibits ALAD in mammalian cell cultures and rodents. Furthermore, a marked decrease in total heme content is observed in brains of SA-treated rats [27]. Otherwise, 2-allyl-2-isopropylacetamide (AIA) and 3,5-diethoxycarbonyl-1,4-dihydrocollidine (DDC) induce a series of enzymatic disruptions which are considered to resemble variegate porphyria [28]. Fasting induces ALAS1 expression via the peroxisome proliferator-activated receptor *γ* coactivator 1*α* (PGC-1*α*) [29]. Griseofulvin is a well-known inducer of APAs and heme depletion [30,31]. Direct ALA administration has also been extensively utilized as a resource to study AHP in vitro and in vivo [32,33,34,35].

Some animal models for studying the three most common AHPs (AIP, HCP, and VP) are currently available [36], in particular, T1/T2 mice have residual 30% HMBS activity and are supposed to partially resemble the pathology of human AIP [37,38]. Quite recently, homozygous *Hmbs* knock-in mice have been produced that display 5% of normal HMBS activity and mimic the exceedingly rare homozygous dominant form of Acute Intermittent Porphyria [39].

## 2. *δ*-Aminolevulinic Acid Toxicity

### 2.1. Mechanisms of Transport and Uptake of δ-ALA in the CNS

When considering the hypothesis of a direct damaging effect of ALA to the brain, some issues arise regarding the possibility of ALA to effectively reach neurotoxic concentrations in the CNS under physiologic conditions.

In vivo studies have shown that the influx-rate constant for ALA movement from bloodstream to the brain is, in fact, quite low and decreases with age. Moreover, it seems to be a result of passive diffusion, except at the CSF-blood and choroid plexus-blood barriers, where the active transport of ALA has been described [40]. This is likely due to the presence of the di- and tripeptide transporter PEPT2 [40,41]. In the choroid plexus, ALA concentrations are considerably high compared to the rest of the brain. Additionally, PEPT2 is expressed at the apical (CNS-facing) side of choroid cells [42], where ALA uptake is seven-fold greater than at the basolateral (blood-facing) side [40]; thus, under physiologic conditions, the choroid plexus is likely to effectively drain whatever ALA might diffuse to the CNS. Both PEPT2 mRNA and protein expression have been localized in subependymal cells, ependymal cells, the choroid plexus [43,44], and, notably, in rat dorsal root ganglia satellite cells (but not in neurons) [44], where also ALA accumulation was described in earlier reports [45].

When incubated with high ALA concentrations (4.0 mM), rat cerebral cortex particles accumulate ALA intracellularly and ALAD activity is enhanced, while HMBS acts as a secondary control step, leading to a build-up of PBG [32,33]. More recently, in mice receiving ALA intraperitoneally, ALA accumulation in the encephalon was demonstrated, together with a plethora of effects on brain metabolism (a reduction of brain ALAS mRNA levels, an increase in cerebellar and hippocampal heme oxygenase activity, an increase in acetylcholinesterase activity, and alterations in factors involved in the management of oxidative stress) [34].

In neonatal rat astrocytes, PEPT2 is likely to be the main transporter of ALA; dipeptides, *α*-amino-containing cephalosporins, and a less acidic pH all negatively affect its transport rate [46]. Moreover, PEPT2 expression in astrocytes appears to decrease with age [47].

Following ALA administration, PEPT2-deficient mice showed substantially lower ALA concentrations in the choroid plexus, cerebral cortex, kidney, eye, blood, and plasma (suggestive of a greater renal clearance), but a five-fold greater concentration in CSF, reaching an eight-fold greater CSF/blood concentration ratio. PEPT2 null mice also displayed a much higher level of susceptibility to ALA toxicity and developed neuromuscular dysfunction following ALA chronic administration; notably, in this study, ALA plasma levels, calculated as the area under the curve, were comparable to those observed in porphyric patients during APAs [48].

In a population of AIP patients, homozygous carriers of a PEPT2 variant with a higher affinity for ALA (PEPT2*1*1) were independently associated with worse renal function and a more severe annual decrease in eGFR, compared to heterozygous or homozygous carriers of a variant with lower affinity (PEPT2*1*2 and PEPT2*2*2) [49]. Conversely, the presence of the PEPT2*2*2 variant was associated with poorer motor dexterity and working memory in children, in the context of low-level lead exposure [50]. Following these pieces of evidence, it has been conjectured that PEPT2 polymorphisms as well as (reversible) functional impairment may act as a modifying factor in defining the penetrance of the AHP phenotype, or even the timing of porphyric attacks [48].

It should be noted that ALA is a substrate for uptake by members of the neurotransmitter sodium and chloride dependent transporter family, whose substrate specificity is, normally, suited to GABA or GABA-like substances such as taurine and β-alanine [51]. In vitro evidence of ALA uptake was found for the transporters SLC6A6, SLC6A13 (whose affinity for ALA was suggested also through homology modeling [52]), possibly SLC6A8 [53], SLC15A1, and SLC36A1 [54]. The latter, in particular, is present in most parts of the human gastroenteric tract, peaking in expression in the small bowel; its mRNA is also detected in the blood-nerve barrier transcriptome [55,56]. These findings, perhaps, should be kept in mind when one thinks of the heightened levels of ALA detected in the peripheral, compared to the central, nervous system [5,44], or of the spasmodic effect exerted by ALA on rat small-bowel preparations [57].

Two final findings are worth mentioning. First, it is interesting the case report of an 82-year-old man who developed a clinical and biochemical picture suggestive of variegate porphyria, immediately following an oral loading of ALA, administered as a prodrug in the context of a photodynamic therapy for Barrett’s esophagitis is interesting [58]. In this case, even allowing for a genetic predisposition of the patient, which may have precipitated a full porphyric syndrome, an -at least initial- direct neurotoxic effect of ALA is too compelling a hypothesis to be easily discarded. Second, it has been shown that *Trypanosoma cruzi’s* epimastigotes produce and excrete substantial amounts of ALA in their culture medium [59]. *Trypanosoma cruzi* is a parasite that constitutively lacks some cytosolic enzymes of the heme biosynthetic pathway [60] and relies on host heme to survive; it is the causative agent of Chagas disease, whose manifestations include, intriguingly, neuropathy and autonomic dysfunction [61,62,63].

### 2.2. Endogenous Production of δ-ALA in the CNS

In principle, in the presence of an enzyme dysfunction in heme biosynthesis, rather than being obtained from the bloodstream, ALA could also be endogenously overproduced by the induction of neuronal (or glial) ALA synthase. Objections against this argument have been posited due to the evidence that liver transplantation (LT) is curative in AHPs [64], with most patients who undergo LT also showing a significant improvement in chronic neurological symptoms. Conversely, it has been reported that patients who received liver grafts from AHP donors within “domino” procedures began to suffer from APAs [65]. Nonetheless, several studies have been conducted to ascertain the possibility of an endogenous brain production of ALA.

In mice, brain mitochondrial ALAS activity is very low at birth, it reaches a peak at about 15 days and then declines steadily during the first 12 months of age [66,67]. In vivo, it seems to be unaffected by fasting, ethanol, AIA, DDC, or barbiturates [66,68,69], whereas it decreases after the administration of cycloheximide or large doses of ALA, or its methyl ester [67]. In fact, brain ALAS mRNA levels in mice were shown to diminish following a chronic or acute intraperitoneal administration of ALA [34]. Notably, injected hematin and CoCl_2_ are not taken up by the brain in vivo and do not affect brain ALAS activity, contrary to the liver isoform [67]. Brain ALAS requires a much lower glycine concentration to reach maximum activity compared to liver, adrenal mitochondrial or heart mitochondrial isoforms [66], but its activity was found to be only 20% compared to the liver isoform. This should still suffice to support the brain’s own requirement for synthesis and turnover of its hemoproteins [67].

Quite interestingly, it has been shown that cerebellar ALAS does undergo upregulation when heme synthesis is disrupted by the intraventricular injection of SA in rats [68], although no changes were observed when SA was administered intraperitoneally [70].

More recently, SA-induced *Alas1* upregulation, inhibited by heme administration, was also observed in vitro in primary cortical neuron cultures [71]. Furthermore, isoflurane and sevoflurane administration increased brain ALAS activity (60% and 163%, respectively) in T1/T2 female mice; sevoflurane was effective also on T1 female mice (550% ALAS activity induction) [69]. In this study, although brain ALAS activity was enhanced, no ALA accumulation, nor variations of ALAS protein expression were detected.

Intriguingly, homozygous *Hmbs* knock-in mice show markedly elevated concentrations of ALA and PBG in the whole CNS (i.e., cerebrum, cerebellum, upper brain stem, and lower brain stem), including the spinal cord, and in the CSF [39]. Differently from T1/T2 mice, *Hmbs* knock-in mice do not show an immediate porphyric biochemical response to porphyrinogenic stimuli. However, they develop a severe neuropathy which closely resembles homozygous dominant acute intermittent porphyria, thus reinforcing the hypothesis of direct neurotoxicity exerted by locally produced ALA (and possibly PBG) [39].

### 2.3. Oxidative Damage, Mitochondrial Alterations and Effects on Iron Homeostasis

From a biochemical perspective, ALA undergoes a phosphate-catalyzed auto-enolization and becomes an oxidizing agent, reacting with iron and *O*_2_ to produce superoxide anion (*O*_2_·), *HO*· radical, and ALA radical (*ALA*·); the latter, in turn, reduces iron and yields the oxidant species dioxovaleric acid (DOVA), by reacting with oxygen [72,73].

Oxidative damage is the main mechanism by which ALA is deemed to cause mitochondrial dysfunction. In vitro, ALA induces mitochondrial swelling and the loss of transmembrane potential [74], possibly due to ROS-driven thiol cross-linking, which may lead to the aggregation of giant pore-like proteins [75]. Interestingly, calcium chelators and (only in the initial phases) catalase and dithiothreitol were able to restore transmembrane potential [74,75]. In addition, in ALA-treated HepG2 cells, an increased expression of mitochondrial biogenesis-related factors and mitochondrial network disruption was observed [76]. Likewise, ALA treatment was shown to alter mitochondrial polarity in rat Schwann cells [35]. ALA-induced lipid peroxidation has also been observed in vitro in rat astrocytes [77], rat Schwann cells [35] and, notably, in cardiolipin-rich liposomes, with a significant increase in their permeability. More specifically, phosphatidylcholine and cardiolipin (a major component of inner mitochondrial membranes) seem to be particularly susceptible to ALA-driven oxidative damage, even when ALA concentrations are in the micromolar range [73]; this has been proposed as an alternative slow-acting (i.e., with a time scale of hours) mechanism contributing to mitochondrial damage [73].

The high susceptibility of myelin-producing cells to oxidation could also play a pathogenic role. It has been shown that rat Schwann cells cultures, when incubated with ALA, suffer a dose-dependent reduction of proteins involved both in the initial stages of myelin formation, as well as in myelin sheath maintenance; decreased levels of sphingomyelins, phosphatidylcholines, and lysophosphatidylcholines were measured accordingly. At the same time, increased levels of carbonylated proteins and peroxidated lipids were detected, suggesting the activation of oxidative events [35].

ALA-driven oxidation is also supposed to exert a disruptive effect on iron homeostasis. Incubation with ALA alters the secondary and tertiary structure of apoferritin (possibly due to selective oxidative damage in tryptophan and cysteine moieties) and impairs its iron uptake ability (which is dependent on L subunits), while keeping its ferroxidase activity intact (dependent on H subunits) [72]. Additionally, ALA-induced iron release from ferritin has been observed in vitro [78]. In previously fasted succinyl acetone methyl ester (SAME)-treated rats, significant ALA-driven increases of total non-heme iron, lipid peroxidation, and of the antioxidant copper zinc superoxide dismutase (CuZnSOD) activity were detected in the brain [79].

Similar results were obtained by intraperitoneal injections of ALA, which caused an increase in total non-heme iron and ferritin in the cortex, in ferritin content in the striatum, in CuZnSOD activity in brain homogenates, lipid peroxidation and protein carbonylation in synaptic membrane preparations of total brain tissue, and in calcium uptake by cortical synaptosomes [80].

The iron regulatory protein 1 (IRP1) is a post-transcriptional regulator that binds to specific mRNA stem-loop structures known as iron-responsive elements (IRE); IREs are present in the mRNA untranslated regions (UTRs) of several proteins pivotal to iron homeostasis. It is therefore worth noting that incubation with ALA or SAME leads to an increased activity of IRP1, which is preventable by the addition of the cell-permeable antioxidant N-acetylcysteine (but not extracellular-acting catalase or superoxide dismutase) [81]. The authors of this study conclude that intracellular ALA should be numbered amongst the co-sensors in the regulation of iron homeostasis.

### 2.4. Neurotransmitter Balance Disruption

The chemical structure of ALA, a five-carbon-chain *ω*-aminoacid, shares some similarities with those of neurotransmitters such as GABA or glutamate. Seminal studies showed that ALA acts as an agonist at presynaptic GABA*_A_* autoreceptors (GABA*_A_*R), displacing GABA and tampering the depolarization-induced GABA release from preloaded nerve endings [82,83]. Prolonged intraperitoneal treatment with ALA in rodents resulted in a decreased binding of ^3^H-muscimol (a potent, selective GABA*_A_*R agonist) in total brain membrane preparations, a result that has been confirmed in vitro in synaptic membranes [80]. Moreover, in vitro and in vivo evidence was provided that the density of GABA*_A_*R decreases in the presence of ALA and DOVA, possibly due to selective oxidative damage (i.e., unlike broad peroxidation) [84]; in this setting, physiologic neurodevelopment could be impaired, since morphologic changes (such as a reduction in the average length of cytoplasmic processes) have been described in P19 cells [84], a cell line which represents a well-established model for studying neuronal differentiation. ALA could also have some GABA mimetic effect on pinealocytes, decreasing melatonin production (see Section 3.3.1) [85].

It is important to acknowledge that, in clinical practice, gabapentinoids are generally viewed as safe, non-porphyrinogenic drugs for the treatment of seizures and control of neuropathic pain in AIP patients [86].

Intracerebral injections of substantial amounts of ALA are known to produce body asymmetry and convulsions in rats, preventable by glutamate receptor antagonists [87]. ALA inhibits glutamate uptake in a dose-dependent, non-competitive, and irreversible manner in rat astrocyte cultures, seemingly because of a disruption of the GLT1 subtype of the glutamate transporter (possibly related, again, to selective oxidation damage) [77]; it also non-competitively impairs glutamate uptake in synaptosomes [82].

With regard to cholinergic neurotransmission, mice receiving intraperitoneal injections of ALA showed an increase in cortex acetylcholinesterase activity after chronic treatment, whereas a reduction in cortical and an increase in hippocampal butyrylcholinesterase activity was observed after an acute treatment [34].

### 2.5. Other Effects of δ-Aminolevulinic Acid

Classical studies on rabbit brain microsomes and chick embryo neuronal cell cultures have shown that ALA exerts an inhibitory effect on Na^+^/K^+^ ATPase [88,89]. In rat cerebellar membranes, ALA impaired signal transduction by lowering the production of the intracellular second messenger cAMP, by a mechanism possibly involving direct oxidative damage to adenylate cyclase [90].

With regard to ALA and sugar metabolism, it is interesting to note that rat cerebral cortex particles displayed an increased glucose uptake (about 145%) when incubated with 2.4 mM ALA in the first hour, subsequently decreasing to around half the control values after 5 h [32]. Other studies on cerebellum particles observed a reduced glucose uptake following ALA administration (87% during 1 h incubation) [33].

## 3. Heme Deficiency-Induced Dysfunction

### 3.1. Alterations in Heme-Dependent Signal Transduction

Growing evidence is emerging in support of heme functioning as a key regulatory and signaling molecule, playing essential roles in the viability of neurons. SA-induced heme deficiency leads to impairments in nerve growth factor (NGF)-induced neuronal differentiation via the early disruption of gene expression [91,92]. In NGF-induced PC12 mature cells (rat pheochromocytoma clonal cells, resembling cells of neural crest origin and a standard model for studying neural development in vitro) SA causes caspase-dependent apoptosis, the activation of the pro-apoptotic c-Jun N-terminal kinase (JNK) and the inhibition of the pro-survival Ras-ERK 1/2 signaling pathways, with downstream inhibitory effects on the gene expression of some regulators (including p53, c-myc, PI3K, Ras, MAPK, JAK1, and MEKK1), and structural proteins (such as SVOP, NCAM, and NPY, and survival motor neuron protein), as well as the upregulation of several stress-induced genes (such as Hsp70, Hsp27, GLUT1, and transferrin receptor) [93]. Notably, mice that lack NPY expression were found to be much more susceptible to seizures [94]. The inactivation of ERK1/2 is likely linked to hypophosphorylation and the reversibly reduced expression of the N-Methyl-d-aspartate receptor (NMDAR) caused by heme depletion, with morphologic changes and neurite loss as a final result [71,95].

### 3.2. Cytochrome Dysfunction

In addition to oxidative damage, mitochondrial failure in AHP could also be driven by cytochrome dysfunction due to heme depletion. Of note, heme is yielded in mitochondria: following an initial series of reactions in the cytosol, coproporphyrin III is imported in mitochondria by ABCB6, a homodimeric porphyrin transporter located in the outer mitochondrial membrane, to undergo the final steps of heme biosynthesis [96]. Several alterations in brain oxidative phosphorylation have been reported in T1/T2 mice, with an increase of Complex II activity in the basal state, and a significant reduction of all four complexes after treatment with phenobarbital, compared to wild-type controls [97]. Recently, a pilot study found an altered mitochondrial bioenergetic profile in AHP patients with moderate-to-severe symptoms, who had a significantly lower oxygen consumption rate at the basal and maximal state, compared to controls and AHP patients with mild or no symptoms [98].

Somewhat unrelated to neuronal damage, it has been observed that a liver isoenzyme (CYP2A5) of the P450 cytochrome family suffers a heme-reversible inhibition in activity and mRNA levels in T1/T2 mice challenged with phenobarbital [99].

### 3.3. Effects on Tryptophan and Glucose Metabolism

Tryptophan 2,3-dioxygenase (TDO, formerly known as tryptophan pyrrolase) is a cytosolic hemoprotein that plays a rate-limiting role in tryptophan degradation [100]. When inhibited in the liver, plasma tryptophan build-up occurs, with augmented tryptophan brain uptake [101] and, reasonably, enhanced serotonin (5-HT) and 5-hydroxyindoleacetic acid (5-HIAA) synthesis. Early studies showed that heme-depleted rats present a dramatic reduction of hepatic TDO activity and an associated increased concentration of brain tryptophan, 5-HT, and 5-HIAA, which was almost completely reversed by parenteral administration of heme [102]. Interestingly, some authors have identified a resemblance between the neurovisceral manifestations of AHP and serotonin syndrome [102].

On the other hand, however, a combined treatment of AIA and DDC was shown to significantly impact the tryptophan metabolic pathway in rat liver in another direction: while serotonin levels decreased and tryptophan concentration rose, an unexpected dose-dependent boost of TDO activity and a reduction of saturation (holoenzyme/apoenzyme ratio) were noted, together with a dose-dependent inhibition of phosphoenolpyruvate carboxykinase (PEPCK) activity [103]. Thus, it has been speculated that the depletion of heme (and possibly pyridoxal phosphate -see below) by these porphyrinogenic drugs may lead to an enhanced substrate-mediated activity of TDO and a switch from the serotonin to the kynurenine pathway, with an increased conversion of tryptophan to kynurenine (whose formation is increased in porphyric animals [103]) and quinolinate, which is an inhibitor of PEPCK [103]. While urinary metabolome studies in AIP patients did not confirm any differences from controls in the serotoninergic route of tryptophan metabolism, the kynurenine pathway was significantly altered with higher urinary concentrations of kynurenine and its metabolites, thus, indirectly confirming an activation of TDO [104]. Notably, PEPCK is a key enzyme in the gluconeogenetic pathway, and the occurrence of its inhibition in a porphyric setting could help to elucidate the role played by glucose in the pathogenesis of AHP. Gluconeogenesis impairment, in the form of PEPCK [28,105] or glycogen phosphorylase (GP) [28] inhibition, has been demonstrated in rats variously treated with porphyrinogenic drugs (AIA, DDC, phenobarbital, or others).

Regarding glucose utilization, ^18^F-FDG PET scans of fasted T1/T2 mice showed a reduced glucose cerebral uptake in the primary somatosensorial and neocortex areas, which reverted to the control values after *Hmbs*-liver gene delivery. Additionally, increased levels of liver and serum ketone bodies, increased hepatic glycogen storage, and reduced pyruvate, lactate, and alanine were recorded, suggestive of a different metabolic response to fasting compared to control or *Hmbs*-liver gene supplied T1/T2 mice [106].

Finally, it is worth mentioning that xanthurenic acid, a product of kynurenine metabolism, has shown potent inhibitory effects on sepiapterin reductase, an enzyme involved in tetrahydrobiopterin (BH_4_) biosynthesis [107]. Tetrahydrobiopterin acts as a cofactor in the hydroxylation of tyrosine, phenylalanine, and tryptophan. An increase of its levels, as a consequence of impaired xanthurenic acid production, has been linked to hyperalgesia [108]. On the other hand, the increased activity of TDO, such as that observed in the aforementioned studies, or a higher production of xanthurenic acid ([109,110], see Section 4.2), could have an impact on tetrahydrobiopterin availability, with some effect, for instance, on nitric oxide biosynthesis ([111], see Section 3.4).

#### 3.3.1. Effect on Melatonin and Circadian Cycles

Interestingly, it has been evidenced that melatonin, a neurohormone with antioxidizing properties and a derivative of serotonin, is able to revert some of the porphyrinogenic toxicity induced by ALA and DDC [112]. AIP patients are known to have decreased levels of plasma melatonin [113], a finding which was confirmed in ALA-treated animal models and in pinealocytes cultures [85]. The decrease in melatonin production has been attributed by some to GABA-like activity exerted by ALA on pinealocytes (whose vascular supply stands outside the BBB) [85].

In mammals, some of the most important factors involved in circadian oscillations depend on heme for their functions: Clock is a hemeprotein with gas-sensor properties, which forms complexes with NO and CO [114]; human Period-2 (hPer2) stability is regulated by heme, which acts as a regulatory ligand [115]; the nuclear hormone receptors (NHRs), REV-ERB*α* and REV-ERB*b* bind heme to regulate their negative action on transcription of their target genes [116,117,118]. Thus, it has been conjectured that heme depletion could be a factor that contributes to circadian disturbances in porphyria: in fact, mice fed with griseofulvin, a well-known inducer of porphyria and a heme depleter [30,31], have a shorter circadian period (as measured on the rhythm of core body temperature levels) phase advances in diurnal rhythm, which were all reversible upon heme supplementation [119].

### 3.4. Effects on Nitric Oxide Synthase

From a clinical and radiological perspective, some CNS manifestations of porphyria have been likened to a picture of posterior reversible encephalopathy syndrome (PRES) [10,11]. The physiopathologic mechanism underlying PRES is generally considered to involve, together with endothelial dysfunction, a reduction of vasodilatory nitric oxide [10,120]. Nitric oxide synthases are hemoproteins, for which the prosthetic heme group is –at least in the neuronal form (nNOS)- a requisite for the dimerization of the enzyme subunits and the correct binding of substrates [121]; interestingly, they also require tetrahydro-biopteroate as a cofactor, and relative BH_4_ deficiency has been linked to uncoupled reactions and the excessive production of the highly reactive species peroxinitrite [111]. NOS is also found throughout the neuronal populations of the myenteric plexus [122], where its dysfunction causes a number of dysautonomic gastrointestinal symptoms, as has been described for patients with nocturnal paroxysmal hemoglobinuria and sickle cell anemia [123,124]. Thus, an impairment in NOS activity following acute or acute-on-chronic heme depletion has been proposed as an explanation for the neurovisceral manifestation of AHP [125]. While nNOS, together with brain soluble guanylate cyclase and heme oxygenase 2, was found to maintain normal levels of activity in the brain as a whole in T1/T2 mice [99], subsequent studies in wild-type mice found a decrease of mitochondrial NOS and an increase of (inducible) iNOS glial expression following an acute ALA intraperitoneal injection [34,126]. Rats treated with SA intraperitoneal injections displayed significantly reduced nitrite/nitrate urinary output, soluble guanylate cyclase activity, kidney homogenate NOS activity, and diminished vascular sensitivity to acetylcholine and MAHMA-NONOate, a NO donor, even in the absence of overt cardiovascular dysregulation [127,128].

## 4. Other Proposed Mechanisms of Toxicity

### 4.1. Cataplerosis Induced by Succinyl-CoA Deficiency

Massive consumption of succinyl-CoA, caused by the overactivation of ALAS, has been proposed as a possible mechanism of mitochondrial damage leading to bioenergetic failure. Indeed, succinyl-CoA is needed as a substrate for the tricarboxylic acid cycle, which in turn provides the reduced species NADPH and FADH_2_ to the mitochondrial respiratory chain. Substrate depletion, or cataplerosis, has been observed in hepatocytes, since T1/T1 mice exposed to phenobarbital displayed selectively reduced liver activities of the TCA cycle enzymes citrate synthase, *α*-ketoglutarate dehydrogenase, and succinate dehydrogenase (the last two are directly involved in the synthesis or utilization of succinyl-CoA) [129]. The hypothesis of a reprogramming of the glucose metabolism in hepatic porphyrias is also supported by studies on urinary metabolome: asymptomatic AIP patients, compared to patients with porphyria cutanea tarda, display higher urinary concentrations in glycolytic intermediates such as acetate, citrate and pyruvate [130], thus hinting at a possible specific dysregulation in the balance among glycolysis, TCA cycle, and phosphorylative oxidation in patients with *HMBS* mutations.

### 4.2. Pyridoxal Phosphate Consumption

An interesting hypothesis has been proposed regarding vitamin B6 (functional) deficiency in patients with AHP. ALA synthase requires pyridoxal phosphate to exert its enzymatic activity, so that ALAS over-induction might, theoretically, lead to depletion of vitamin B6.

An early report described a case of an AIP patient whose ALA and PBG urinary excretion decreased following induction of vitamin B6 deficiency, and rose following pyridoxine supplementation [131], corroborating the hypothesis of a pivotal role played by pyridoxal phosphate in the heme biosynthetic pathway. A few years later, a seminal study found significantly lower concentrations of plasma pyridoxal phosphate and significantly higher xanthurenic acid excretion in a group of 21 patients with AIP, compared to an age-matched healthy control group [109]. In a more recent unindexed study, a population of 50 patients with *HMBS* mutations were found to have normal plasma pyridoxal phosphate levels, but a significantly higher urinary 3-hydroxykynurenine/xanthurenic acid ratio, homocysteine and methionine levels compared to controls, suggestive of a functional vitamin B6 deficiency in the AIP group [110]. Of further interest, a poorer vitamin status was detected in a population of AIP and VP patients that concerned vitamin B6, vitamin B12, and red blood cell folates. In this study, symptomatic patients displayed lower vitamin B group levels and a significant association was identified between lower plasma pyridoxal phosphate and a history of recurrent attacks [132]. Previously mentioned urinary metabolome studies also found an impairment in the vitamin B6-mediated conversion of kynurenine to kynurenic acid in AIP patients [104].

Inborn enzymatic dysfunctions, leading to pyridoxal phosphate deficiency, are at the origin of some forms of vitamin B-responsive epilepsies (pyridoxine dependent epilepsy and pyridoxal phosphate dependent epilepsy) [133,134,135]. Moreover, toxicity due to isoniazid, an antitubercular drug that impairs vitamin B6 metabolism, appears to cause nerve lesions and a clinical picture comparable to those found in AIP [136,137]. Thus, it might be speculated that pyridoxal phosphate may also play a role in defining the neurologic clinical picture of patients with AHP.

## 5. Conclusions

**Figure 1:**
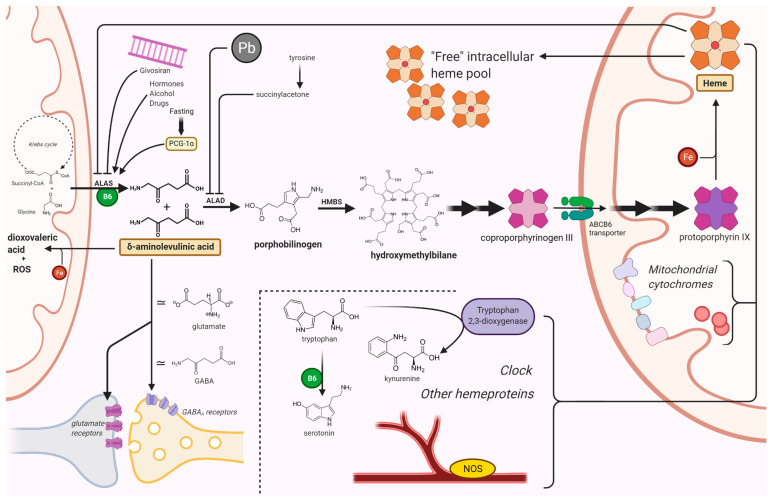
**Mechanisms of neuronal damage in acute hepatic porphyrias**. ALAS condenses glycine and succinyl-CoA into ALA, in the first step of the biosynthesis of heme. After an initial series of reactions in the cytosol, coproporphyrin III is imported in mitochondria by ABCB6, a homodimeric porphyrin transporter located in the outer mitochondrial membrane. ALAS is induced by porphyrinogenic stimuli (e.g., fasting, alcohol, or certain drugs) which supposedly induce an increased metabolic demand for heme. In particular, fasting induces ALAS1 expression via the peroxisome proliferator-activated receptor *γ* coactivator 1*α* (PGC-1*α*). Lead and succinyl acetone cause a porphyria-like picture since they inhibit ALAD. Acute intermittent porphyria, the most common AHP, is an autosomal dominant disease caused by an abnormal function of HMBS. ALA and (in most cases) PBG accumulate in patients with acute porphyrias during neurovisceral attacks. Givosiran, a siRNA-based drug for the treatment of AHPs, acts by impairing ALAS mRNA translation in the liver. Among other toxic effects, ALA undergoes auto-enolization to yield the highly reactive dioxovaleric acid (DOVA) and other oxidant species; it also interferes with GABA and glutamate receptors. Lack of heme has pleiotropic effects on cytochromes, nitric oxide synthases, tryptophan 2,3-dioxygenase, and several other hemeprotein; it may also affect the regulatory functions of the intracellular “free” heme pool. Pyridoxal phosphate figures among the factors involved in this highly connected network of reactions. Other possible mechanisms of neuronal damage are described in the text. ABCB6, ATP-binding cassette transporter B6; ALA, *δ*-aminolevulinic acid; ALAD, ALA dehydratase; ALAS, ALA synthase; B6, pyridoxal phosphate; Fe, iron; GABA, *γ*-aminobutyric acid; HMBS, hydroxymethylbilane synthase; NOS, nitric oxide synthase; Pb, lead. Created with BioRender.com (last accessed date: 22 November 2021).

The pathway leading to the biosynthesis of heme is inscribed into a complex network of interactions that also includes some of the most fundamental processes of basal metabolism. Therefore, a disruption in any of the steps of this pathway is likely to interfere in a pleiotropic fashion with the viability of cells and tissues, thus giving rise to multiple possible mechanisms of pathogenesis (Figure 1). Here, we have endeavored to outline the (sometimes contradictory) evidence that has been gathered to date, regarding the mechanisms of neurotoxicity in acute hepatic porphyrias.

Porphyric patients suffer a considerable burden of disease [138], with both debilitating chronic symptoms and the always lurking menace of life-threatening porphyric attacks. Successfully recognizing an acute hepatic porphyria represents a major diagnostic challenge for the clinician [139,140]. Until recently, therapeutic options consisted of the treatment of acute attacks with parenteral heme arginate and glucose infusions, avoidance of triggering factors, and—in extreme cases—liver transplantation [141,142].

A promising new drug, givosiran, has provided potential avenues as a new effective weapon in the armamentarium of the clinician [143]. Givosiran is a small interfering RNA (siRNA) that targets ALAS mRNA in hepatocytes, exploiting the RNA-induced silencing complex (RISC) to impair its translation. Even though some adverse events, possibly related to induction of relative heme deficiency [144,145,146] and partly reversible [144,147], have been recorded, givosiran has proved to be very effective in reducing the biochemical markers of disease, lowering the mean annualized rate of porphyric attacks, and improving the quality of life of patients [143].

In our opinion, this does not abate the need to clarify the mechanisms of pathogenesis in AHPs. On the contrary, the encouraging perspective offered by these therapeutic advancements raises the stakes for answering the unsolved questions posed by such a fascinating and challenging field of research.

## 6. Acronyms

**5-HIAA** 5-hydroxyindoleacetic acid. **5-HT** 5-hydroxytryptamine (or serotonin). **ABCB6** ATP-binding cassette transporter B6. **ADP** ALA dehydratase deficiency porphyria. **AHP** acute hepatic porphyrias. **AIA** 2-allyl-2-isopropylacetamide. **AIP** acute intermittent porphyria. **ALA** aminolevulinic acid. **ALAD** aminolevulinic acid-dehydratase. **ALAS** aminolevulinic acid synthase. **BH**_4_ tetrahydrobiopteroate. **CNS** Central Nervous System. **CSF** cerebro-spinal fluid. **CuZnSOD** copper-zinc superoxide dismutase. **DDC** **3:5**-diethoxycarbonyl-1,4-dihydrocollidine. **DOVA** dioxovaleric acid. **HCP** hereditary coproporphyria. **HMBS** hydroxymethylbilane-synthase or porphobilinogen-deaminase (PBGD). **IRE** iron-responsive element. **IRP1** iron regulatory protein 1. **JNK(s)** c-Jun N-terminal kinase(s). **LT** liver transplantation. **MAHMA-NONOate** methylamine hexamethylene methylamine NONOate. **NGF** nerve growth factor. **nNOS** neuronal nitric oxyde synthase. **PBG** porphobilinogen. **PC12 (cells)** rat pheochromocytoma clonal cells. **PEPCK** phosphoenolpyruvate carboxykinase. **PRES** Posterior reversible encephalopathy syndrome. **SA** succinylacetone. **SAME** succinylacetone methyl esther. **TCA** tricarboxylic acid. **TDO** **tryptophan 2:3**-dioxygenase. **UTR** untranslated region. **VP** variegate porphyria.
